# Improved rates of exclusive breastfeeding at 14 weeks of age in KwaZulu Natal, South Africa: what are the challenges now?

**DOI:** 10.1186/s12889-018-5657-5

**Published:** 2018-06-19

**Authors:** C. Horwood, L. Haskins, IM Engebretsen, S. Phakathi, C. Connolly, A. Coutsoudis, L. Spies

**Affiliations:** 1 Centre for Rural Health, University of KwaZulu-Natal, Durban, South Africa; 2 Centre for International Health, Department of global public health and primary care, University of Bergen, Bergen, Norway; 3 Department of Paediatrics & Child Health, School of Clinical Medicine, Nelson R Mandela School of Medicine, University of KwaZulu-Natal , Durban, South Africa; 4 KwaZulu-Natal Department of Health, Pietermaritzburg, KwaZulu-Natal South Africa

**Keywords:** Exclusive breastfeeding, Breastfeeding, Risk factors, HIV infection, South Africa

## Abstract

**Background:**

Increasing the rate of exclusive breastfeeding (EBF) to 50% in the first six months of life is one of six major global targets set by the United Nations Decade of Nutrition, and is essential to achieve the sustainable development goals to eradicate hunger and end malnutrition by 2030.

**Methods:**

A survey using multistage random sampling design included 99 primary health care (PHC) clinics in all 11 districts in KwaZulu-Natal (KZN). All mothers and caregivers of infants 14 weeks of age attending the clinics in the study period were requested to participate in a structured interview to explore feeding practices since birth. As non-maternal caregivers may not have detailed knowledge of feeding practices, they provided limited information about current feeding practices. Respondents who consistently reported giving no other food or fluids except breastmilk since birth were classified as practising exclusive breastfeeding (EBF), and those who were currently breastfeeding but had given other food or fluids since birth were categorised as practising mixed breastfeeding (MBF).

**Results:**

A total of 4172 interviews were conducted with mothers and caregivers of 14 week old infants. Among mothers 49.8% were EBF, 23.1% were MBF and 27.0% were not breastfeeding. Among non-maternal caregivers 11.8% reported EBF, 23.4% MBF and 62.3% were not giving breastmilk. Higher education (OR 0.6, 95% CI 0.4–0.8) and being in the highest socio-economic tertile (OR 0.7, 95% CI 0.6–0.9) were risk factors for not practising EBF, while returning to work (OR 0.3, 95% CI 0.2–0.3) or school (OR 0.2 95% CI, 0.1–0.3) was associated with less EBF. HIV-positive mothers were more likely to have never started breastfeeding (OR 3.6, 95% CI 2.7–4.8). However, they were similar in having stopped breastfeeding by 14 weeks (OR 1.1, 95% CI 0.9–1.4) compared to HIV-negative mothers, and, they had similar rates of EBF at 14 weeks of age (OR 1.0, 95% CI 0.9–1.3).

**Conclusions:**

Estimates of breastfeeding practices at 14 weeks in KZN are higher than previously shown. However, particular challenges that should be addressed if international targets for EBF are to be achieved include improving breastfeeding practices of HIV positive mothers and supporting all mothers, particularly working or schooling mothers to continue giving breastmilk while they are away from their children.

## Background

Increasing the rate of exclusive breastfeeding (EBF) in the first six months of life to 50% is one of six major global targets set by the United Nations (UN) Decade of Nutrition [1], and improving exclusive and sustained breastfeeding rates is essential for achieving sustainable development goals (SDGs) to eradicate hunger and end malnutrition by 2030 [2]. If implemented globally at near universal levels (> 90%), optimal breastfeeding practices could reduce global child deaths by more than 800,000 [3], which makes breastfeeding the most effective preventive intervention to improve infant mortality [4]. The numerous benefits of breastfeeding continue to accrue throughout childhood and into adulthood, including reduced morbidity from diarrhoeal and respiratory illness [5], and reduced risk of overweight and obesity in children, another of the six UN targets [1, 6]. Although long term follow-up studies on infant feeding practices have implicit methodological challenges [7], the favourable effect of breastfeeding on cognitive performance indicators has recently been presented [8, 9]. In addition, mothers benefit from breastfeeding as longer duration of breastfeeding substantially reduce risk of breast and ovarian cancer [10]. Although investing in interventions to promote and support breastfeeding comes with a cost, this cost should be balanced against reduced mortality and morbidity and increased productivity, which generate short and long-term financial benefits [11].

WHO infant and young child feeding guidelines recommend early initiation of breastfeeding within one hour of birth, EBF for the first six months, followed by sustained breastfeeding for two years and beyond, with introduction of nutritionally-adequate and safe complementary feeding from six months of age [12]. Despite increasing recognition of the benefits of breastfeeding across all settings and income levels, breastfeeding rates remain low in many countries. Rates of EBF among children under 6 months in low- and middle- income countries was estimated at 37% in 2013, resulting in substantial lost benefits for mothers and children [11]. In South Africa (SA), EBF rates among infants aged under 6 months was found to be 31.6% in the most recent Demographic and Health survey conducted in 2016 [13], which demonstrates substantial improvements from the previous estimate of 7% in 2003 [14]. However, early cessation of breastfeeding, mixed feeding and addition of complementary feeds in the first six months, remains the norm among South African mothers [15, 16], and there are few valid estimates of age-specific breastfeeding rates.

A number of factors have been identified as barriers to exclusive and sustained breastfeeding, including sociocultural, health system, community and individual factors [11]. Lack of maternal self-efficacy, poor experiences of breastfeeding and perceptions of insufficient milk are important individual-level barriers to breastfeeding [11, 16]. Provision of breastfeeding support from health workers, community health workers, peer supporters and others in the family or community have consistently been associated with improved feeding practices [11, 17, 18]. Returning to work after the birth of the infant is a major barrier to breastfeeding and is significantly associated with lower rates of breastfeeding, earlier cessation of breastfeeding, and mothers planning to return to work are less likely to plan to EBF [19–21].

In addition, South Africa has some of the highest rates of HIV infection in the world, with 29.7% of women attending government antenatal clinics testing HIV positive in 2013, this is highest in KwaZulu Natal province (KZN) where antenatal HIV seroprevalence was 40.1% in 2013 [22]. In the past decade frequent changes to the HIV and infant feeding guidelines has resulted in the promotion of changing and, at times, contradictory infant feeding messages, some of which were not supportive of breastfeeding [23]. It was not until 2016 that HIV and infant feeding guidelines were revised, so that current feeding advice for HIV exposed infants is similar to that for all infants [24].

In this paper we report the findings of a large survey of infant feeding practises at 14 weeks of age among all infants attending the clinics in KwaZulu-Natal, South Africa and describe determinants of any breastfeeding and EBF at 14 weeks of age in our setting.

## Methods

A quantitative survey was conducted among infant mother/caregiver pairs attending primary health care (PHC) clinics in all 11 districts in KZN. Immunisation coverage in KZN is high with 89.9% children fully immunised at one year in 2014/15 [25], thus this sample could be considered similar to the whole population of infants at this age [26].

All 536 fixed and mobile PHC clinics in KZN were included in the sampling frame. Health facilities in each district were selected for inclusion in the study using multistage stratified random sampling. All caregivers aged 15 years or above attending participating clinics with an infant aged 13- < 16 weeks (91–111 days) were eligible to participate during the inclusion period, including maternal and non-maternal caregivers. Since non-maternal caregivers could not be expected to have accurate information about the mother’s situation, they were asked a small number of questions regarding current feeding practices only. Trained field workers used a tablet-based data collection system to collect data from all eligible caregivers.

### Sample size

Since estimates at the district level were wanted, the sample size requirement was stratified by district, and the primary sampling unit was the clinic. In the first stage, nine clinics were randomly selected from a list of all clinics providing immunisations in KZN, in each of 11 districts (total 99 clinics). The sample size was calculated using the formula, *n* = Z2pq/d2 (where Z = 1.96 at 95% confidence; *p* = proportion who are exclusively breastfeeding, *q* = 1-p; d = absolute precision). For this study, we presumed that *p* < = 0.4 based on previous routine data and literature; *q* = 0.6; precision (d) = +/− 5%. This yielded a required sample size of 369 per district. The final sample size required was thus 369*11 = 4059 respondents.

In the second stage of sampling all eligible mothers attending the clinic within the study period were approached for participation. Data collection continued in all selected clinics in each district contemporaneously until the required sample was realised for that district therefore resulting in a self-weighted sample.

### Data management and analysis

Data were collected using android devices and uploaded to a centralised server in real time using proprietary software. Extensive data quality checks were conducted to ensure data completeness and validity.

The classification of rurality is based on definitions provided by the KZN Department of Cooperative Governance and Traditional Affairs (KZN-COGTA), which segments the KwaZulu Natal province into 5 distinct categories. Clinics in areas A, B1 and B2 (metro’s, secondary cities and large towns) were designated as urban, whereas areas B3 and B4 (small towns and mainly rural) were designated as rural [27].

Mothers were asked questions about feeding practices since birth, including a question about ever having given other food or fluids to the infant, and later follow-up questions specifically asked about whether the infant had ever been given formula milk, and whether any pre-lacteal feeds were given. Only those who were breastfeeding and consistently reported having given no other food or fluids, except breastmilk, were classified as exclusively breastfeeding (EBF). Those who reported currently breastfeeding but who reported giving other food or fluids at any time since birth, were categorised as mixed breastfeeding (MBF). Non-maternal caregivers were categorised as EBF if the carer reported that the infant had never been given any other food or fluids other than breastmilk.

Data processing and analysis was conducted using Stata 13.1 (StataCorp. 2013. Stata Statistical Software: Release 13. College Station, TX: StataCorp LP.) Given the multiple stage sampling design, Stata’s survey settings (“svyset {pweight=wgt], strata (district) psu(clinic)” was used make provincial level estimates and the appropriate 95% confidence intervals (95% CIs). This analysis includes both mothers and non-maternal caregivers presenting data on infant feeding characteristics at 14 weeks, the social and economic circumstances of caregivers as well as local infrastructure. However, due to the limited information available from the non-maternal carers, only the sub-set with mothers was included in the risk factor regression analysis on factors associated with any breastfeeding and EBF.

Logistic regression analysis was done for two dependent variables using unweighted data but adjusted for study design. First, any breastfeeding versus non-breastfeeding, and second EBF versus mixed and non-breastfeeding. This was to see if EBF had a similar associated risk pattern to any breastfeeding in general. Crude and adjusted models are presented. For the adjusted models, factors significant at *p* < 0.2 at the bivariable level or reported in the literature were included in the model. The overall likelihood ratio test (Wald statistic) was assessed for both regression models. For post-hoc model testing, the linktest was used to test the model specification, and the Hosmer-Lemeshow test for goodness-of-fit was used to determine how well the model fit. Linearity was tested using lowess smoothing. The area under the ROC curve for the breastfeeding model was used to determine whether sensitivity and specificity was acceptable.

Principle components analysis (PCA) was used to created socio-economic quintiles. Factors included were household assets and wall material of household (traditional or brick). The PCA scores were categorized into low, medium and high tertiles.

## Results

A total of 4172 interviews were conducted with caregivers attending 78 fixed and 21 mobile clinics in 11 districts of KZN between May 2014 and March 2015. Among the infants 3659/4172 (87.7%) attended with the mother and 513 (12.3%) attended with a carer who was not the mother (Table 1). The target age of the infant was 14 weeks, coinciding with the scheduled 14-week vaccination visit. The median, intra-quartile-range (IQR), age of the child was 102 days (IQR 98–106).

**Table 1:** Characteristics of all participating child carers

	N (4172)	%	95% CI
Relationship with the child			
Mother	3659	87.7	(86.7–88.7)
Grandmother	196	4.7	(4.1–5.4)
Father	36	0.9	(0.6–1.2)
Other relative	203	4.9	(4.3–5.6)
Non relative caregiver	78	1.9	(1.5–2.3)
Population group			
African	4094	98.1	(97.7–98.5)
Coloured	19	0.5	(0.3–0.7)
Indian origin	46	1.1	(0.8–1.5)
White	13	0.3	(0.2–0.54)
Sex of baby (% male)	2045	49.0	(47.5–50.5)
Geographic area (rural)	2845	68.2	(66.8–69.6)

Characteristics of participating mothers are shown in Table 2. The geographical location and population group characteristics were similar to those of all caregivers. The median age of 3659 participating mothers was 24 years (IQR 20–29 years), amongst whom 52 mothers (1.4%) reported that they did not stay in the same house as the infant. Most mothers reported that they were in a relationship with the father of the child 3244/3659 (88.7, 95% CI 87.6–89.6%), and 874/3659 (23.9, 95% CI 22.5–25.3%) mothers were living in the same house as the father of the child. Around half of all fathers had completed high school 1887/3659 (51.6, 95% CI 50.0–53.2%), and more than half of the fathers were employed 2393/3659 (65.4, 95% CI 63.8–66.9) and had provided regular support for the infant since birth, including providing money and other goods 2255/3659 (61.6, 95% CI 60.0–63.2%).

**Table 2:** Characteristics of participating mothers and infants

	*N* = 3659	%	95% CI
Mothers age			
< 20 years	702	19.2	(17.9–20.5)
20–24 years	1191	32.6	(31.0–34.1)
25–29 years	858	23.5	(22.1–24.9)
> 30 years	908	24.8	(23.4–26.2)
Population group			
African	3604	98.5	(98.0–98.8)
Coloured	14	0.4	(0.2–0.6)
Indian origin	30	0.8	(0.6–1.2)
White	11	0.3	(0.2–0.5)
Education			
Primary school or less	324	8.9	(8.0–9.8)
Completed Grades 8 to 11	1884	51.5	(49.9–53.1)
Complete Grade 12 or higher	1451	39.7	(38.1–41.3)
Returned to school since infant was born	246	6.7	(6.0–7.6)
Work			
Mother has done no paid work in past 12 months	2779	75.9	(74.5–77.3)
Mother has done paid work in past 12 months but not since infant was born	584	16.0	(14.8–17.2)
Mother has done paid work since infant was born	296	8.1	(7.2–9.0)
Financial support (multiple sources)			
Mother receives child support grant for any child	2445	66.8	(65.3–68.3)
Mother receives maternity grant/pay from employer	137	3.7	(3.2–4.4)
Mother receives disability grant	22	0.6	(0.4–0.9)
Mother receives maintenance/money from partner	2456	67.1	(65.6–68.6)
Mother receives money from family members	1196	32.7	(31.2–34.2)
Household geographic area (rural)	2498	68.3	(66.7–69.8)
Has more than one child	2010	54.9	(53.3–56.5)
Sex of infant (% male)	1802	49.2	(47.6–50.9)

### Contact with the health service

Although most participating mothers attended antenatal clinics 3554/3659 (97.1%), some mothers 610/3554 (17.1%) reported that they did not receive any infant feeding advice during their antenatal clinic visits. Similarly, although most participating mothers delivered their infant in a health facility 3494/3659 (95.5%), some did not receive breast feeding advice while at the facility 791/3494 (22.6%). Most mothers reported that the infant was placed skin-to-skin after delivery 2404/3569 (65.7%), but less than half 1587/3659 (43.4%) of the mothers reported that they had initiated breastfeeding within one hour of delivery.

In regards to community based services, 391/3659 (9.4%) mothers reported receiving a visit from a community health worker (CHW) during pregnancy, and 453/3659 (10.9%) mothers reported receiving a visit from a CHW after delivery.

### Infant feeding practices

Reported current feeding practices among all participants, both non-maternal caregivers and mothers are shown in Table 3. Among 513 non-maternal caregivers, 154 (35.3%_wgt_, 95% CI_adj_: 28.7–42.4) reported that the infant was currently receiving breastmilk. Of those, 85/154 (39.9%_wgt_, 95% CI _sfj_ 26.0–55.6) non-maternal caregivers reported feeding the infant with expressed breastmilk during the clinic visit. Most mothers, 2705/3659 (73.0% _wgt_, 95% CI _adj_: 69.5–76.2) reported that they were currently breastfeeding (either mixed breastfeeding or EBF).

**Table 3:** Reported feeding practices among all carers, non-maternal and maternal carers

	Not currently breastfeeding		Mixed breastfeeding		EBF^a^		Unable to assess	
	n	%wgt (95% CI)	n	%wgt (95% CI)	n	%wgt (95% CI)	n	%wgt (95% CI)
Non maternal carers *N* = 513	338	62.3 (55.4–68.6)	81	23.4 (17.6–30.5)	73	11.8 (7.0–19.2)	21	2.5 (1.2–5.0)
Mothers *N* = 3659	954	27.0 (23.8–30.5)	800	23.1 (19.0–27.8)	1905	49.8 (45.0–54.7)	0	
All carers *N* = 4172	1292	31.9 (29.0–35.0)	881	23.2 (19.5–27.3)	1978	44.6 (40.2–49.1)	21	0.3 (0.2–0.7)

^*a*^ *Exclusive breastfeeding*

### HIV positive mothers

Of the 3659 mothers surveyed, 3567 (97.5%) had been tested for HIV and were able to report their HIV status; 55 mothers chose not to answer the HIV questions; eight mothers had never had an HIV test and data were missing for 29 mothers.

**Fig. 1:**
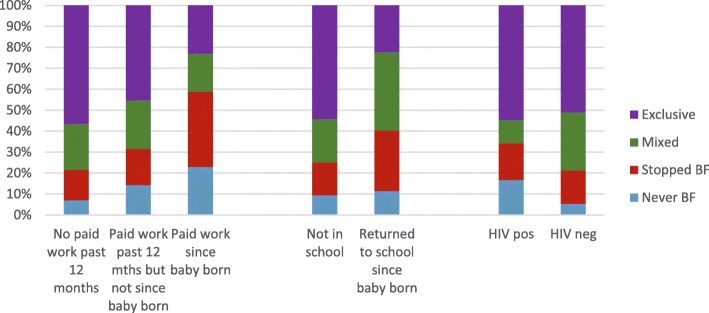
Breastfeeding practices among all mothers according to work status, school return and HIV-status (*N* = 3659)

Of those mothers who reported their HIV status, 1274/3567 (34.8) were HIV positive, of whom 1134 (89.0%) reported currently taking antiretroviral treatment. The bi- and multivariable analysis showed that HIV positive mothers were less likely to be currently breastfeeding (OR 0.5, 95% CI 0.4–0.6) (Table 4) but they had similar rates of EBF at 14 weeks of age (adjusted OR 1.2, 95% CI 0.96–1.4) (Table 5) when compared to HIV negative mothers. HIV positive mothers were far more likely to have never started breastfeeding (OR 3.6, 95% CI 2.7–4.8). They were similar in having stopped breastfeeding at 14 weeks (OR 1.1, 95% CI 0.9–1.4). Feeding practices according to HIV status are shown in Fig. 1.

**Table 4:** Factors associated with any breastfeeding vs non-breastfeeding (non-BF) among participating mothers, *N* = 3659, bi- and multi-variable logistic analysis

	Non-BF		BF		Total	bi variable		multi-variable	
Risk Factors	n	%	n	%	n	OR	95% CI	OR	95% CI
Age of the mother									
< 20	147	20.9	555	79.1	702	ref		ref	
20–29	511	24.9	1538	75.1	2049	**0.8**	**(0.6–0.9)**	0.8	(0.7–1.1)
30–49	296	32.6	612	67.4	908	**0.5**	**(0.4–0.7)**	**0.6**	**(0.5–0.9)**
Education level									
0–7	53	16.4	271	83.6	324	ref		Ref	
8–11	437	23.2	1447	76.8	1884	**0.6**	**(0.5–0.9)**	**0.7**	**(0.5–0.9)**
12	460	32.0	977	68.0	1437	**0.4**	**(0.3–0.6)**	**0.4**	**(0.3–0.6)**
Geographic area									
Rural	573	22.9	1925	77.1	2498	ref		ref	
Urban	381	32.8	780	67.2	1161	**0.6**	**(0.5–0.8)**	0.8	(0.7–1.0)
Household connected to electricity									
No	118	16.0	618	84.0	736	ref		ref	
Yes	835	28.6	2087	71.4	2922	**0.5**	**(0.4–0.6)**	**0.7**	**(0.5–0.9)**
Socioeconomic quintile									
1 (Low)	223	18.3	997	81.7	1220	ref		ref	
2	283	23.2	937	76.8	1220	**0.7**	**(0.6–0.9)**	1.0	(0.8–1.3)
3 (high)	448	36.8	771	63.2	1219	**0.4**	**(0.3–0.5)**	**0.7**	**(0.5–0.9)**
Mother working									
No/never	780	23.2	2583	76.8	3363	ref		Ref	
Returned work	174	58.8	122	41.2	296	**0.2**	**(0.2–0.3)**	**0.3**	**(0.2–0.4)**
Mother returned to school since birth									
No/not in school	855	25.1	2558	74.9	3413	ref		ref	
Yes	99	40.2	147	59.8	246	**0.5**	**(0.4–0.7)**	**0.2**	**(0.2–0.3)**
HIV status									
Negative	488	21.3	1805	78.7	2293	ref		ref	
Positive	435	34.1	839	65.9	1274	**0.5**	**(0.4–0.6)**	**0.5**	**(0.4–0.6)**
Unknown	31	33.7	61	66.3	92	**0.5**	**(0.3–0.9)**	0.7	(0.4–1.0)
Feeding advice given at ANC clinic									
No	189	31.0	421	69.0	610	ref		ref	
Yes	765	25.1	2284	74.9	3049	**1.3**	**(1.1–1.7)**	**1.4**	**(1.1–1.8)**
Feeding advice given at delivery									
No	206	26.0	585	74.0	791	ref		ref	
Yes	748	26.1	2120	73.9	2868	1.0	(0.8–1.2)	0.8	(0.7–1.0)
Infant placed skin-to-skin after delivery									
No	347	27.6	908	72.4	1255	ref		ref	
Yes	607	25.2	1797	74.8	2404	1.1	(0.9–1.4)	0.98	(0.8–1.2)
Initiated breastfeeding within 1 h of delivery									
No	644	31.1	1428	68.9	2072	ref		ref	
Yes	310	19.5	1277	80.5	1587	**1.9**	**(1.5–2.2)**	**1.7**	**(1.5–2.1)**
Mother helped with breastfeeding initiation									
No	558	30.9	1250	69.1	1808	ref			
Yes	396	21.4	1455	78.6	1851	**1.6**	**(1.4–2.0)**	**1.4**	**(1.2–1.7)**
Feeding advice by CHW: antenatal									
No	903	27.2	2417	72.8	3320	ref		ref	
Yes	51	15.0	288	85.0	339	**2.1**	**(1.5–2.9)**	**1.9**	**(1.3–2.7)**
Feeding advice by CHW: postnatal									
No	878	26.8	2403	73.2	3281	ref		ref	
Yes	76	20.1	302	79.9	378	**1.5**	**(1.1–1.9)**	1.0	(0.7–1.5)

*Significant associations shown in bold text*

**Table 5:** Factors associated with not exclusive breastfeeding (non-EBF) versus exclusive breastfeeding (EBF) practice among participating mothers, *N* = 3659, bi- and multi-variable logistic analysis

	Non-EBF		EBF		Total	Bi variable		Multi variable	
Risk Factors	n	%	n	%	n	OR	95% CI	OR	95% CI
Age of the mother									
< 20	374	53.3	328	46.7	702	ref		ref	
20–29	951	46.4	1098	53.6	2049	**1.3**	**(1.1–1.6)**	1.1	(0.9–1.4)
30–49	429	47.2	479	52.8	908	**1.3**	**(1.0–1.6)**	1.1	(0.8–1.4)
Education level									
Grades 0–7	114	35.2	210	64.8	324	ref		ref	
Grades 8–11	882	46.8	1002	53.2	1884	**0.6**	**(0.5–0.8)**	0.7	(0.6–1.0)
Grade 12 (completed school)	758	52.2	693	47.8	1451	**0.5**	**(0.4–0.7)**	**0.6**	**(0.4–0.8)**
Geographic area									
Rural	1103	44.2	1395	55.8	2498	ref		ref	
Urban	651	56.1	510	43.9	1161	**0.6**	**(0.4–0.9)**	0.8	(0.6–1.0)
Household connected to electricity									
No	287	39.0	449	61.0	736	ref		ref	
Yes	1466	50.2	1456	49.8	2922	**0.6**	**(0.5–0.8)**	0.9	(0.7–1.2)
Socioeconomic tertile									
1 (Low)	483	39.6	737	60.4	1220	ref		ref	
2	572	46.9	648	53.1	1220	**0.7**	**(0.6–0.9)**	0.9	(0.7–1.1)
3 (high)	699	57.3	520	42.7	1219	**0.5**	**(0.4–0.6)**	**0.7**	**(0.6–0.9)**
Mother working									
No/never	1526	45.4	1837	54.6	3363	ref		ref	
Returned to work	228	77.0	68	23.0	296	**0.2**	**(0.2–0.3)**	**0.3**	**(0.2–0.3)**
Mother returned to school since birth									
No/not in school	1563	45.8	1850	54.2	3413	ref		ref	
Yes	191	77.6	55	22.4	246	**0.2**	**(0.2–0.3)**	**0.2**	**(0.1–0.3)**
HIV status									
Negative	1123	49.0	1170	51.0	2293	ref		ref	
Positive	577	45.3	697	54.7	1274	1.2	(0.96–1.4)	1.0	(0.9–1.3)
Unknown	54	58.7	38	41.3	92	0.7	(0.4–1.0)	0.8	(0.5–1.3)
Feeding advice given at ANC clinic									
No	358	58.7	252	41.3	610	ref		ref	
Yes	1396	45.8	1653	54.2	3049	**1.7**	**(1.3–2.2)**	**1.5**	**(1.2–2.0)**
Feeding advice given at hospital									
No	408	51.6	383	48.4	791	ref		ref	
Yes	1346	46.9	1522	53.1	2868	1.2	(0.98–1.5)	0.9	(0.7–1.1)
Infant placed skin-to-skin after delivery									
No	668	53.2	587	46.8	1255	ref		ref	
Yes	1086	45.2	1318	54.8	2404	**1.4**	**(1.1–1.7)**	**1.3**	**(1.0–1.5)**
Initiation of breastfeeding within one hour of delivery									
No	1094	52.8	978	47.2	2072	ref		ref	
Yes	660	41.6	927	58.4	1587	**1.6**	**(1.3–1.9)**	**1.4**	**(1.2–1.7)**
Mother helped with initiation of breastfeeding									
No	919	50.8	889	49.2	1808	ref		ref	
Yes	835	45.1	1016	54.9	1851	**1.3**	**(1.0–1.5)**	1.2	(0.95–1.4)
Feeding advice by CHW: antenatal									
No	1636	49.3	1684	50.7	3320	ref		ref	
Yes	118	34.8	221	65.2	339	**1.8**	**(1.5–2.3)**	**1.4**	**(1.1–1.8)**
Feeding advice by CHW: postnatal									
No	1607	49.0	1674	51.0	3281	ref		ref	
Yes	147	38.9	231	61.1	378	**1.5**	**(1.2–1.9)**	1.1	(0.8–1.6)

Probability of EBF adjusted of study design only (unweighted). Significant associations shown in bold text

### Returning to work or school

Feeding practises among mothers who had returned to work or school at the time of the interview are shown in Fig. 1.

### Factors associated with any breastfeeding at 14 weeks

According to a multi-variable analysis the following factors were associated with not breastfeeding at week 14: older mothers, mothers with more education, urban mothers, richer households, including those with electricity, having returned to work and school, and being HIV positive or having an unknown HIV status (Table 4). Antenatal care advice in the clinics as well as by community health workers were protective factors, and early initiation of breastfeeding and breastfeeding assistance and support were also identified as protective factors for any breastfeeding at 14 weeks.

### Factors associated with exclusive breastfeeding

There was a similar pattern of risk- and protective factors shown for exclusive breastfeeding versus any other feeding as was seen as for any breastfeeding in general (Table 5), where higher education and being in the highest socio-economic tertile were risk factors for not exclusively breastfeeding. However, returning to work (OR 0.3, 95% CI 0.2–0.3) and school (OR 0.2 95% CI, 0.1–0.3) were most strongly associated with less exclusive breastfeeding. Breastfeeding advice at the antenatal care clinics came out most strongly (OR 1.5, 95% CI 1.2–2.0) followed by advice in the communities as well as early initiation of breastfeeding and assistance with initiating breastfeeding as protective factors. Skin-to-skin contact after delivery was a protective factor for exclusive breastfeeding (OR 1.3, 95% CI 1.0–1.5), rather than for any breastfeeding (Tables 4 and 5).

## Discussion

This study provided estimates of feeding practices at 14 weeks of age among infants attending a random selection of clinics in KwaZulu-Natal, South Africa, including those attending with non-maternal caregivers and with mothers. Further, it presents factors associated with any breastfeeding and exclusive breastfeeding among mothers, and shows that working or schooling mothers and those who are HIV infected have quite different feeding practices compared to other mothers. The survey design included a feeding recall since birth, which allowed for categorisation of infant feeding practices up to the interview time point into non-breastfeeding, mixed breastfeeding and exclusive breastfeeding. The study did not consider “lapses” and/or “switches” of feeding category, so information on any feeding behaviour since birth accumulated to the current feeding category. This is the strictest definition we have of exclusive breastfeeding [28, 29], meaning that no other feeds had been given up to the time of interview other than breast milk and prescribed medicines, drops or syrups [30]. Most previous published reports of EBF rates in SA have been based on a 24 h or seven day feeding recall, which is a less strict definition of exclusive breastfeeding [31] or report rates of breastfeeding among all children under 6 months [32]. The rigorous methodology employed in our study suggests that exclusive breastfeeding until 14 weeks of age has improved substantially in KZN, a trend that is supported by other recently reported data from across South Africa [31, 33, 34]. This may suggest that interventions to support breastfeeding, including the Tshwane declaration made by the South African Department of Health in 2011 [35] and changes to infant feeding recommendations for HIV infected women that support breastfeeding, may have resulted in improvements to breastfeeding rates. Although these results are encouraging, further improvements are still required.

The multi-variable regression analysis identified key risk-and protective factors associated with the respective feeding categories. The most striking finding was that being older, having higher education, going back to work or school, and being in a higher socioeconomic tertile was associated with less breastfeeding at 14 weeks, and that this was just as important as being HIV-positive in determining non-breastfeeding. Thus, a key message from this study, is that there is a need to focus on the general hurdles for breastfeeding per se as well as continuing to provide the important support for women with an HIV-positive status.

This study demonstrated clearly that a key challenge to sustaining breastfeeding was when the mother returns to work or school. While WHO has re-emphasized the importance of supporting breastfeeding in the workplace, and returning to work has been shown to be a barrier to optimal breastfeeding practices in many settings [36–39], there remains little evidence that this is translated into public policies at national levels. Only 42 countries among 185 surveyed complied with the recommendations of the International Labour Organisation for a minimum of 18 weeks maternity leave [40]. Breastfeeding can be continued after return to work if supportive policies are in place, including childcare or support for expressing of breastmilk [40, 41], and reducing barriers to breastfeeding can reduce absenteeism and improve motivation among workers [41]. In South Africa, the Basic Conditions of Employment Act provides for four consecutive months of maternity leave but there are no other provisions in the act to support breastfeeding [42].

There are major practical challenges to achieving sustained breastfeeding, particularly exclusive breastfeeding, during work or school and mothers may require considerable support [43, 44]. These challenges include developing skills in expressing breastmilk ahead of the return to work or school, safe storage of expressed breast milk at home and at work, and cup-feeding. It is important for health workers, including CHWs, to be confident in supporting this process. Of note was that many participating mothers in this study reported giving their babies expressed breastmilk at some time when they were away from the infant, which suggests that expressing breastmilk is being practiced in these communities. Almost half of working women in South Africa are working in the informal sector, including domestic workers. These women do not have access to social protection, and are frequently vulnerable and low-paid. More information is needed about informally working  women and how breastfeeding could be supported in this setting.

Almost universal attendance at health facilities provides opportunities for health workers to provide appropriate counselling, but many mothers reported having not received feeding advice during their visits, leading to missed opportunities to reinforce key infant feeding counselling messages. Our data suggests that whenever advice is given, this is effective at improving feeding practices, and this observation is supported by other studies [45]. It is crucial that mothers attending the clinic for antenatal care or with an infant, receive infant feeding counselling at every visit. In the postnatal period advice should not just include generic counselling messages but should be individually based and targeted at providing mothers with support for dealing with day-to-day infant feeding challenges [46]. Closing the gaps in feeding advice and assistance at clinic and community level should be a key focus to improve breastfeeding rates.

Our data shows that receiving a CHW visit is effective in improving breastfeeding practices, and this is supported by other studies [18, 47]. Home visits allow accessible, appropriate, individualised breastfeeding support to be given, and may involve family members if appropriate. Studies suggest that family members and social support of mothers at the household level plays a strong role in influencing feeding practices, so that CHWs have an important role to play in providing context specific feeding advice and support for mothers experiencing problems sustaining breastfeeding [18]. However, very few mothers in this study reported having received a visit from a CHW, although over 11,000 CHWs are employed in KZN to provide services at household level, and a core function of these CHWs is to visit pregnant women and new mothers in their homes, including providing breastfeeding support. The role of CHWs in provision of maternal and child health services needs to be strengthened and closer supervision and mentoring may be necessary.

HIV positive status remains a key determinant of mothers’ choosing not to breastfeed. However, the message of avoiding mixed feeding has largely been adhered to by HIV infected mothers. Thus, we found similar rates of exclusive breastfeeding in the HIV positive group as in the HIV negative group, despite there being more non-breastfeeding in the HIV positive group. The most recent WHO HIV and infant feeding guidelines recommend breastfeeding for all, irrespective of HIV status, together with support for anti-retroviral drug adherence among HIV infected women [48]. There is a need for continued breastfeeding promotion and support among HIV positive women in the light of these new guidelines, which have been adopted in South Africa [48]. A key message that should be promoted is that if EBF cannot be achieved for some mothers and babies, it is still important to sustain as much breastfeeding as possible. This message applies to HIV infected mothers, as long as the mother is compliant with ART and is virally suppressed. This is an area where confusion arises as this is contrary to the strong message provided previously that mixed breastfeeding was unacceptable for HIV infected mothers and the breastfeeding period should be short. This study showed that the ‘no mixed feeding message’ has been strongly received for HIV positive mothers, but a concern is that fear of mixed feeding could lead HIV positive mothers to either abstain from initiating breastfeeding or stop breastfeeding prematurely. In addition, only 87% of mothers reporting taking anti-retroviral treatment, highlighting the importance of ART adherence. This means that in this setting there might be a need also in parallel to strengthen the infant-HIV-prophylaxis treatment as an alternative if the mother have delayed PMTCT participation or is non-adherent [49].

### Strengths and limitations

Major strengths were that this study was a large, representative sample of KwaZulu-Natal, a large province in South Africa. Given the high attendance for 14 week immunisation visits, this study provides a good estimate of population level feeding practices in KZN. Inclusion of information from non-maternal caregivers, a group often is excluded in infant feeding studies, added additional strength. The survey design with its comprehensive instruments enabled a risk factor analysis.

The limitations of the study was having a 14 week recall period which can provide information bias as mothers may have had difficulty recalling exactly what her infant was fed. Although we expected limited social desirability recall bias as the research team was independent from the health providers, this can never be eliminated, especially not at facility based studies. A possible limitation was that we assumed there was no design effect at sample size calculation, however the analysis was adjusted for clustering at clinic level.

## Conclusion

This survey from KZN, South Africa highlights an improvement in exclusive breastfeeding practices at 14 weeks of age. Particular challenges to achieving optimal breastfeeding practices at 14 weeks, include the need to improve breastfeeding practices among HIV positive mothers and supporting working or schooling mother to continue giving breastmilk while they are away from their child. However, it is important to note that breastfeeding practices among all mothers are sub-optimal and there is a need to strengthen individualised feeding counselling at the various contact points for pregnant and lactating mothers. In the effort to improve breastfeeding practices for HIV positive women, there is also a need to consider the gap in PMTCT services.

## Abbreviations

ART: Ante-retroviral therapy
BREC: Biomedical Research Ethics Committee
CHW: Community health worker
CI: Confidence interval
EBF: Exclusive breastfeeding
HIV: Human Immunodeficiency Virus
IQR: Inter quartile range
KZN: KwaZulu-Natal
KZN-COGTA: KwaZulu-Natal Department of Cooperative Governance and Traditional Affairs
MBF: Mixed breastfeeding
OR: Odds Ratio
PCA: Principal component analysis
PHC: Primary health care
PMTCT: Prevention of mother-to-child HIV transmission
SA: South Africa
SDG: Sustainable development goals
UKZN: University of KwaZulu-Natal
UN: United Nations
Wgt: Weighted
WHO: World health organization
